# Management and complications of complete heart block in pregnancy

**DOI:** 10.1177/1753495X211033489

**Published:** 2021-10-01

**Authors:** AP Christensen, V Singh, AJ England, R Khiani, AS Herrey

**Affiliations:** 1 William Harvey Research Institute, Queen Mary University of London, London, UK; 2 Department of Obstetrics & Gynaecology, Royal Free Hospital NHS Foundation Trust, London, UK; 3 Department of Anaesthesia, Royal Free Hospital NHS Foundation Trust, London, UK; 4 Department of Cardiology, Royal Free Hospital NHS Foundation Trust, London, UK; 5 Department of Cardiology, Bartshealth NHS Trust, London, UK

**Keywords:** Heart block, pacing, heart failure, pacemaker, pregnancy

## Abstract

Although rare, increasing numbers of women with pacemakers are becoming pregnant. We describe the complications of a woman with arrhythmia and a pacemaker for complete heart block experienced before, during, between and after her pregnancies. We illustrate the benefits of multidisciplinary care, good communication and regular assessment in a stable, but complex woman.

## Introduction

Complete heart block in the young is rare, but often well tolerated in pregnancy unless associated with structural heart disease, in which case can carry significant morbidity and mortality.^1^ Cardiac output is usually maintained by increasing stroke volume, but some people require additional management when facing physiological challenges such as pregnancy. The decision to implant a pacemaker in a young asymptomatic is never taken lightly because of the risk of long-term adverse events such as infection, venous access problems and sequelae of cardiac stimulation at non-optimal sites. Predicting adverse events in pregnancy is particularly difficult when heart block is associated with structural disease, and published evidence is limited. This case report describes two pregnancies in a woman with congenital heart disease who required cardiac pacing.

## History, investigations and treatment

**Figure 1.**
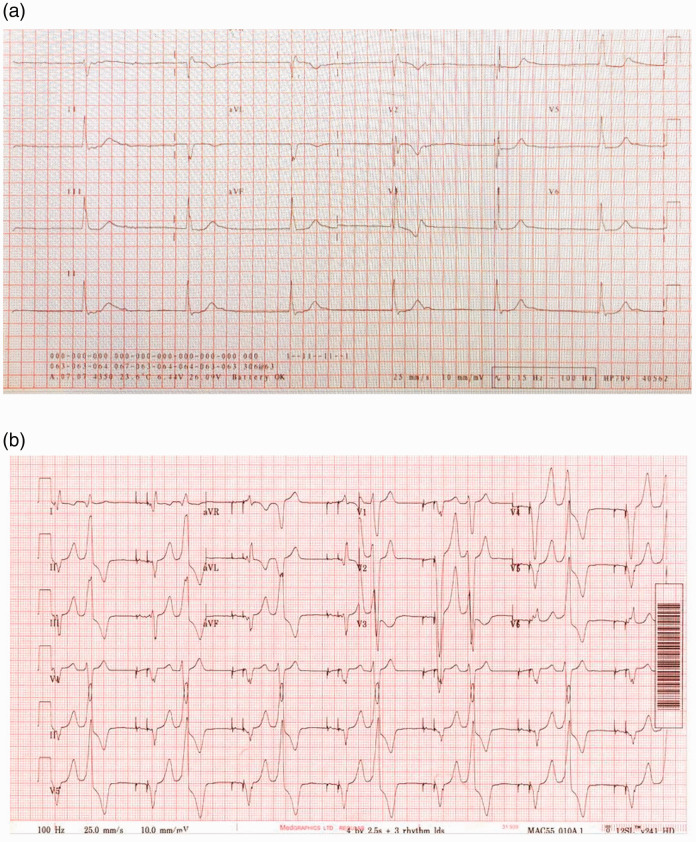
12-Lead ECG showing a paced bigeminal rhythm. (a) 12-lead ECG showing complete AV block with RBBB escape. (b) 12-lead ECG showing a paced bigeminal rhythm.

The woman had congenital atrial septal defect, pulmonary stenosis and heart block. She underwent pulmonary valvotomy and atrio-septal defect repair aged three years but was lost to follow-up. She represented with chest pain aged 27 and was found to be in complete atrioventricular block with intermittent junctional rhythm and right bundle branch block (RBBB) escape, indicative of both sinus node and atrioventricular (AV) node disease (Figure 1(a)). Her echocardiogram showed right ventricular (RV) dilatation and severe pulmonary regurgitation. The same year, following preconception counselling, a dual chamber pacemaker was implanted to enable an increase in cardiac output in preparation for pregnancy and labour. Her ventricular bigeminy (Figure 1(b)) was treated with bisoprolol.

A first trimester echo found a dyssychronous left ventricle (LV) with preserved function and pulmonary regurgitation. These were well tolerated, and routine antenatal care was continued, albeit with frequent clinic reviews in the joint maternal clinic.

At 38 weeks’ gestation, she presented with prolonged rupture of membranes. Labour was induced with prostaglandin, augmented with oxytocin and she received an epidural for pain control. During labour she felt unwell. Her electrocardiogram showed ventricular bigeminy with a rate of 80 bpm, but a pulse oximeter found a peripheral pulse of 40 bpm, which was the same as the maternal heart rate record on the cardiotocogram (CTG). The pacemaker model had an accelerometer-driven output increase algorithm and also recorded a rate of 80 bpm. It had failed to detect the increased demands of labour in a woman who was bed bound with an epidural. After re-programming from demand to fixed rate pacing, initially 80 bpm, then 100 bpm, a significant improvement in woman symptoms and suppression of bigeminy was seen.

After 8 h of labour, she underwent caesarean section for failure to progress. Following delivery, the pacemaker was reprogrammed to demand pacing at 60 bpm and the postpartum period was uneventful.

Four years later, she booked her second pregnancy. She was asymptomatic, and her exercise tolerance was maintained with no orthopnoea or paroxysmal nocturnal dyspnoea. She was taking bisoprolol 2.5 mg a day. A routine echocardiogram unexpectedly found severe LV impairment with severe septal dyssynchrony from left bundle branch block caused by right ventricular pacing and subsequent development of asymptomatic left ventricular impairment. She was started on tinzaparin at 28 weeks to reduce her risk of thromboembolism and remained asymptomatic with unchanged LV function at 32 weeks.

She underwent elective caesarean section at 36 weeks due to severely impaired LV function, with concomitant insertion of an intrauterine contraceptive device, under regional anaesthesia with invasive monitoring. In the postpartum period, enalapril was added to the bisoprolol.

Her LV systolic dysfunction persisted at six months postpartum, possibly secondary to chronic RV pacing. Her pacemaker was upgraded to a cardiac resynchronisation device.^2^ A defibrillator was not inserted due to the expected functional improvement with cardiac resynchronisation therapy. Three months later, her LV function had improved, but not normalised, probably due to a high number of ventricular ectopic beats impairing the attempted biventricular pacing. On her most recent pacing check, however, she was bi-ventricular paced 94% of the time with significant reduction of her QRS duration and her ventricular ectopic burden had reduced to 4%. Further improvement of her LV function is therefore expected.

## Discussion

Rate responsive pacemakers reproduce the physiological rate response of the sino-atrial node. The most common algorithms used to determine rate are accelerometers which detect forward movement, and minute ventilation sensors that monitor respiratory rate.

During pregnancy, cardiac output normally increases by up to 50% and in labour the cardiac output increases by a further 15% in the first stage and 50% in the second. An autotransfusion occurs with each uterine contraction with 300 ml to 500 ml of blood re-entering the circulation.^3^ Therefore, some women with cardiac conditions are unable to achieve this. Small case series show that some women with complete atrioventricular block and structurally normal hearts can cope with the haemodynamic changes of labour without pacing.^4^,^5^ Our patient had structural cardiac abnormalities, a predictor of worse outcome.^1^ Her cardiac function was further compromised by arrhythmia and the specifications of her pacemaker. Arrhythmias, such as a bigeminy, are very common in pregnancy due to the increased physiological demand, increased levels of circulating catecholamines and pregnancy-related enlargement of the cardiac chambers, and most are benign.^6^ However, these can cause complications in women with underlying cardiac conditions. Therefore, women with pre-existing cardiac conditions wishing to become pregnant require preconception counselling to identify risks. This allows them to make informed decisions about pregnancy and reduce complications. In her first pregnancy, the pacemaker relied on accelerometery to increase heart rate. Complications arose in labour for two reasons, (1) inability of her particular pacemaker model to detect the need for increased pacing in labour, and (2) from her underlying heart rhythm of bigeminy only generating a pulse wave with alternate beats, causing a low cardiac output. When pacemaker settings were changed from demand to fixed rate pacing at a higher base rate of 100 bpm, the ventricular ectopic beats were suppressed, and her cardiac output increased. We would therefore suggest in women with permanent pacemakers which rely on accelerometry-driven detection of increased demand (especially when having epidurals), increasing the base rate in labour to compensate for the increased cardiac demand, which may otherwise not be detected by the pacemaker. This is less relevant for pacemakers relying on a combination of accelerometry and minute ventilation to adapt the rate.

The development of pacing induced cardiomyopathy with severe LV impairment before her second pregnancy was an unfortunate, but well-described complication of long-term pacing in the young, which responded to resynchronisation therapy.^7^,^8^

This case highlights the benefit of multidisciplinary teams looking after pregnant women with cardiovascular disease. The ESC guidelines on the management of cardiovascular disease in pregnancy recognise this and emphasise the value of a pregnancy heart team for all women at moderate or high risk of complications during pregnancy.^9^

## References

[1] Hidaka N Chiba Y Fukushima K , et al. Pregnant women with complete atrioventricular block: perinatal risks and review of management. Pacing Clin Electrophysiol 2011; 34: 1161–1176. 21797903 10.1111/j.1540-8159.2011.03177.x

[2] ESC Guidelines on cardiac pacing and cardiac resynchronization therapy. Eur Heart J 2013; 34: 2281–2329. 23801822 10.1093/eurheartj/eht150

[3] Nelson-Piercy C (2015). Handbook of Obstetric Medicine. 5th ed. CRC Press. 10.1201/b18316

[4] Keepanasseril A, Maurya DK, Suriya Y and Selvaraj R. Complete atrioventricular block in pregnancy: report of seven pregnancies in a patient without pacemaker. *BMJ Case Rep*. 2015, bcr2014208618, 9 March 2015. Doi:10.1136/bcr-2014-208618. 10.1136/bcr-2014-208618 PMC4369012 25754166

[5] Hidaka N Chiba Y Kurita T , et al. Is intrapartum temporary pacing required for women with complete atrioventricular block? BJOG 2006; 113: 605–607. 16579804 10.1111/j.1471-0528.2006.00888.x

[6] Adamson DL Nelson-Piercy C. Managing palpitations and arrhythmias during pregnancy. Heart 2007; 93: 1630–1636. 18003696 10.1136/hrt.2006.098822 PMC2095764

[7] Tantengco MV Thomas RL Karpawich PP. Left ventricular dysfunction after long-term right ventricular apical pacing in the young. J Am Coll Cardiol 2001; 37: 2093–2100. 11419893 10.1016/s0735-1097(01)01302-x

[8] Sarkar NC Tilkar M Jain S , et al. Evaluation of long term effect of RV apical pacing on global LV function by echocardiography. J Clin Diagn Res 2016; 10: OC03–6. 27134910 10.7860/JCDR/2016/18547.7397 PMC4843296

[9] European Society of G, Association for European Paediatric C, German Society for Gender M, et al. ESC Guidelines on the management of cardiovascular diseases during pregnancy: the Task Force on the Management of Cardiovascular Diseases during Pregnancy of the European Society of Cardiology (ESC). Eur Heart J 2011; 32: 3147–3197. 21873418 10.1093/eurheartj/ehr218

